# A High-Resolution, Wide-Swath SAR Imaging System Based on Tandem SAR Satellites

**DOI:** 10.3390/s22207747

**Published:** 2022-10-12

**Authors:** Liwei Sun, Chunsheng Li

**Affiliations:** School of Electronics and Information Engineering, Beihang University, Beijing 100083, China

**Keywords:** synthetic aperture radar (SAR), high-resolution wide-swath, minimum-energy criterion

## Abstract

For the spaceborne synthetic aperture radar (SAR), it is difficult to obtain high resolution and wide width at the same time. This paper proposes a novel imaging system based on tandem SAR satellites, where one obtains coarse resolution and wide swath by the scanning mode, and the other obtains the undersampled echo from the same swath. The high resolution is achieved by associating the tandem SARs’ echo and using the minimum-energy-based algorithm. Finally, a high-resolution wide-swath SAR system is designed, and its imaging performance is verified by simulated data and real airborne SAR data.

## 1. Introduction

The synthetic aperture radar (SAR) is a powerful sensor that can observe the Earth’s surface day and night, regardless of the weather conditions [1,2,3]. This sensor has been widely adopted in the military and civilian remote sensing areas, such as oceanographic observations, sea-ice monitoring, and vegetation mapping [4,5,6]. For future SAR development, high-resolution wide-swath (HRWS) technology plays an important role in these applications. However, the high resolution and the wide swath contradict each other due to the minimum antenna area constraint [7]. On the one hand, the high resolution requires a high-pulse repetition frequency (PRF) to suppress the Doppler ambiguity. At the same time, the wide swath needs a low PRF to avoid range ambiguity. Therefore, HRWS cannot be obtained in a monostatic SAR system simultaneously [8].

For achieving monostatic HRWS-SAR imaging, researchers proposed many methods, which can be divided into two categories: 1. The first approach focuses on the range ambiguity issue, where a sufficiently high PRF is selected to avoid the Doppler ambiguity. The frequency diversity array (FDA) technique is proposed in [9]. It introduces a small increment across the all array elements, then the echoes received from different ambiguous regions are separated. Moreover, the multiple-elevation-beam technology is adopted in [10]. It employs real-time beamforming on receipt to form multiple narrow elevation beams, each following the radar echo of a different transmitted pulse; thus, the range ambiguity is suppressed. 2. The second approach is to cope with the Doppler ambiguity issue, where a low PRF is chosen to avoid range ambiguity. For example, the azimuth multichannel technology compensates for the lack of temporal sampling by the spatial sampling of points of multiple channels [11]. Thus, the effective sampling rate is increased, and the Doppler ambiguity is suppressed [12]. Those technologies mentioned above can effectively improve the width of the swath while ensuring the azimuth resolution for monostatic SAR. However, they all have a complex structure, leading to greater challenges in designing a large satellite. To reduce hardware complexity [13,14,15], separates the transmitter and receiver of SAR to a multi-satellite network, but the increased launch volume and mass for the deployment of multiple satellites and additional hardware for accurate phase synchronization are indispensable [16]. Furthermore, [17] develops a monostatic SAR−HRWS imaging system based on compressed sensing (CS) theory, but its imaging performance depends on the sparsity of the observed scene.

In order to achieve HRWS-SAR imaging, this paper proposes a novel imaging system based on tandem SAR satellites. The first one works in the scanning mode to obtain the coarse observations of a wide swath. The second one obtains the downsampled echo from the same swath. Then, the high-resolution imaging is achieved by associating the tandem SARs’ echo and using the minimum-energy-based algorithm. Since the precise phase synchronization between two satellites is unnecessary, the system cost can be reduced.

This paper is organized as follows: Section 2 introduces the working modes of the two satellites. Section 3 constructs the signal model and describes the minimum-energy-based algorithm. Section 4 designs an example system and verifies its imaging performance. Finally, Section 5 concludes this paper.

## 2. Imaging System Based on Tandem SAR Satellites

The proposed system consists of two satellites flying successively in the same orbit. The distance between two satellites is far enough to ensure that they do not interfere with each other. Both satellites adopt a scanning mode, but the scanning methods are different. The introduction is presented in detail as follows.

### 2.1. Working Mode of the First Satellite

**Figure 1:**
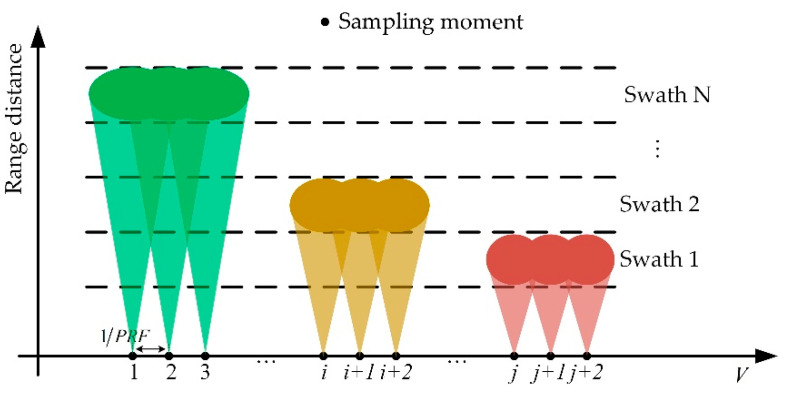
Pulse assignment diagram of the traditional scanning method, where the antenna beam jumps periodically among different subswaths. *N* indicates the number of subswaths.

The first satellite, namely, Sat-1, operates in the traditional scanning mode [18], which adopts the burst mode, and assigns the aperture time to multiple subswaths. It can broaden the range swath at the cost of coarsening the azimuth resolution. The observation geometry of Sat-1 is shown in Figure 1. To achieve an imaging system with azimuth resolution $\delta_{az}$, the necessary burst time $T_{n}$ of the *n*th subswath should satisfy the following:

(1) $$T_{n}=\frac{0.886\cdot \lambda R_{0,n}}{2V\delta_{az}}$$

where λ is the wavelength, $R_{0,n}$ is the mean slant range in the zero Doppler plane of the *n*th subswath, and V is the speed of the SAR platform.

According to the principle of traditional ScanSAR, we have

(2) $$B_{D}=\left(N+1\right)\cdot B_{n}$$

where $B_{n}$ and $B_{D}$ denote the Doppler bandwidth of the *n*th subswath and the 3 dB bandwidth of the azimuth antenna, respectively. According to Equation (2), the coarsening of the azimuth resolution is in the order of *N* + 1, while the range swath compared to a strip-map system is increased by *N*.

### 2.2. Working Mode of the Second Satellite

**Figure 2:**
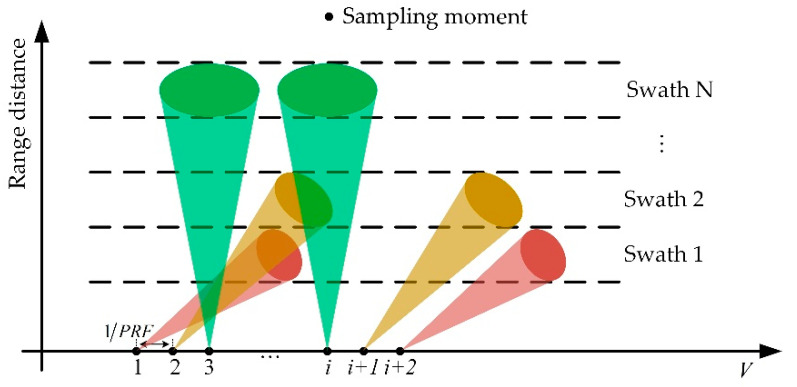
Pulse assignment diagram of the new scanning method, where each pulse is randomly assigned to one of the subswaths on the ground and different subswaths have different squint angles.

The second satellite, which is noted as Sat-2, adopts a new scanning method. It randomly assigns each pulse to one of the subswaths on the ground and receives the reflected echo, as shown in Figure 2.

**Figure 3:**
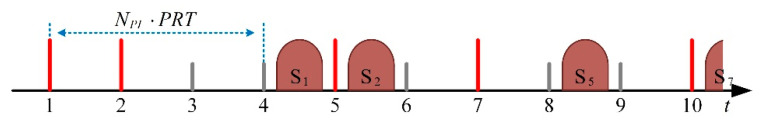
Timing diagram of the subswath’s pulse transmitting and receiving, where the vertical line indicates the pulse emission time, and the long red line indicates that the current pulse is assigned to this subswath. The short gray line suggests that the current pulse is assigned to other subswaths. S_1_, S_2_, and S_5_ represent the reflected echoes of pulses 1, 2, and 5, respectively.

Since spaceborne SAR platforms usually work at an altitude of more than 100 km [19], the reflected echoes of transmitted pulses return to the receiver after several pulse intervals (PIs). Because all subswaths share one receiver, the waiting PI number for different subswaths must be the same to avoid overlapping of the received echoes. Assuming that the waiting PI number is $N_{PI}$, as shown in Figure 3, the reflected echoes of all the subswaths should return to the receiver during the $N_{PI}+1\mathrm{th}$ PI after transmission. To that end, we set the farthest subswath work in broadside mode and the other subswaths in squint mode. The squint angle increases as the distance of subswath to nadir decreases, as shown in Figure 2.

In the trans-receive view, the time interval of the two transmitted pulses is fixed to 1/PRF, while the time interval of the two received pulses of any subswath is not certain, and the average sampling rate is lower than the Nyquist rate. Therefore, the received echoes are downsampled.

### 2.3. Comparison of the Two Scanning Methods

**Figure 4:**
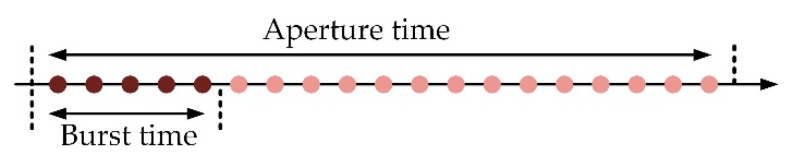
Traditional scanning method.

**Figure 5:**
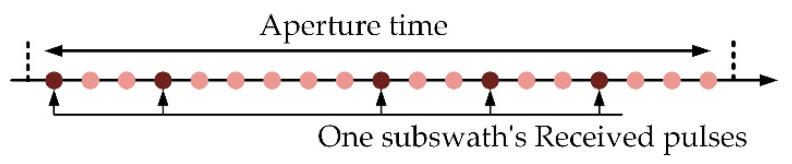
Proposed scanning method.

The scanning method adopted in Sat-2 can keep the entire aperture time of targets, which is different from the traditional method. As shown in Figure 4 and Figure 5, each point represents an azimuth sample in the echo of a ground target and corresponds to a transmitted pulse. The traditional scanning method assigns a continuous pulse train to one subswath, thus truncating the target’s aperture time. The proposed scanning method randomly chooses the pulses assigned to one subswath; it can retain the entire aperture time, although with downsampling.

An example system is designed in Section 4. After the echoes are received, the coarse observations obtained by Sat-1 are used as prior knowledge to achieve high-resolution imaging from the received Sat-2 data.

## 3. Signal Model and Imaging Algorithm

According to the system introduced above, Sat-1 works in traditional scanning mode; its received data can be processed by existing algorithms [1,2,3]. This section mainly focuses on the modeling and imaging for Sat-2.

### 3.1. Signal Model of the Sat-2

According to the SAR principle described in [20], the original SAR echo of Sat-2 can be expressed as

(3) $$S_{0}\left(\eta ,\tau \right)=A_{0}W_{a}\left(\eta -\eta_{c}\right)W_{r}\left(\tau -\frac{2R\left(\eta \right)}{c}\right)\exp\left\{-j\frac{4\pi }{\lambda }R\left(\eta \right)\right\}\cdot \exp\left\{j\pi k_{r}{\left(\tau -\frac{2R\left(\eta \right)}{c}\right)}^{2}\right\}$$

where τ and η denote the range and azimuth time, respectively, and $\eta_{c}$ is the beam center crossing time. R(η) is the slant distance between the observed target and the platform at azimuth time η, c is the speed of light, $k_{r}$ is the pulse modulation frequency, $A_{0}$ is a complex constant, and $W_{a}\left(\cdot \right)$ and $W_{r}\left(\cdot \right)$ are the azimuth and range antenna mode, respectively. *M* is the number of targets on the observed scene.

After range compression, Equation (3) can be expressed as

(4) $$S_{1}\left(\eta ,\tau \right)=A_{0}W_{a}\left(\eta -\eta_{c}\right)\sin c\left[B_{r}\left(\tau -\frac{2R\left(\eta \right)}{c}\right)\right]\exp\left\{-j\frac{4\pi }{\lambda }R\left(\eta \right)\right\}$$

where $B_{r}$ is the bandwidth of the transmitted pulse. $S_{1}\left(\eta ,\tau \right)$ can also be expressed in the matrix form as

(5) $$S_{i}=A\cdot \sigma_{i}$$

where $S_{i}$ and $\sigma_{i}$ are the observation vector and scene vector of the *i*th range gate, respectively. $\sigma_{i}={\left[\sigma_{i,1},\sigma_{i,2},\cdots ,\sigma_{i,K}\right]}^{T}$ and $\sigma_{i,k}$ is the equivalent backscatter coefficient of the *k*th pixel, *K* is the number of pixels in the *i*th range gate. $A=\left[A_{1},A_{2},\cdots ,A_{k},\cdots ,A_{K}\right]$ is the sensing matrix with

$$A_{k}=W_{a}\left(\eta -\eta_{c,k}\right)\cdot \exp\left\{-j\frac{4\pi }{\lambda }R\left(\eta ,k\right)\right\}$$

where $\eta ={\left[\eta_{1},\eta_{2},\cdots ,\eta_{N_{a}}\right]}^{T}$ is the azimuth time vector, $N_{a}$ is the total number of sampling points in the azimuth direction, $\eta_{c,k}$ is the beam center crossing time of the *k*th pixel, and R(η,k) is the slant distance vector between the *k*th pixel and the platform.

According to Equation (5), the traditional imaging algorithms [21,22] based on matched filtering can be expressed as

(6) $${\hat{\sigma }}_{i}=A^{H}\cdot S_{i}$$

where $A^{H}$ is the conjugate transpose matrix of A. Since the received echo of Sat-2 is seriously downsampled, severe ambiguity energy occurs in the imaging result of Equation (6). The CS-based SAR system introduces the sparsity prior to the scene reconstruction to compensate for the information lost by downsampling, and then selects the solution with the best sparsity as the estimation of the observed scene, as shown in (7).

(7) $${\hat{\sigma }}_{i}=\underset{{\hat{\sigma }}_{i}}{\arg\min{\left\|{\hat{\sigma }}_{i}\right\|}_{0}}\;s.t.\;S_{i}=A{\hat{\sigma }}_{i}$$

However, the estimation accuracy of (7) depends on whether the scene is sparse or whether it can be sparsely represented accurately. In order to achieve better imaging performance, this paper modifies the traditional minimum-energy criterion [23] introducing the coarse observation as prior information and constructs the optimization problem as

(8) $${\hat{\sigma }}_{i}=\underset{\hat{\sigma }}{\arg\min}\langle {\hat{\sigma }}_{i},Q^{-1}\cdot {\hat{\sigma }}_{i}\rangle \;s.t.\;S_{i}=A{\hat{\sigma }}_{i}$$

where $Q=\mathrm{diag}\left(\sigma_{d,i}\cdot \sigma_{d,i}^{*}\right)$ is a K×K matrix and $\sigma_{d,i}$ is the amplitude envelope of the imaging result of the *i*th range gate on the ground, which can be obtained by interpolating the imaging result of Sat-1. Finally, the solution with the minimum weighted energy is chosen as the scene estimation.

### 3.2. Minimum-Energy-Based Imaging Algorithm

**Figure 6:**
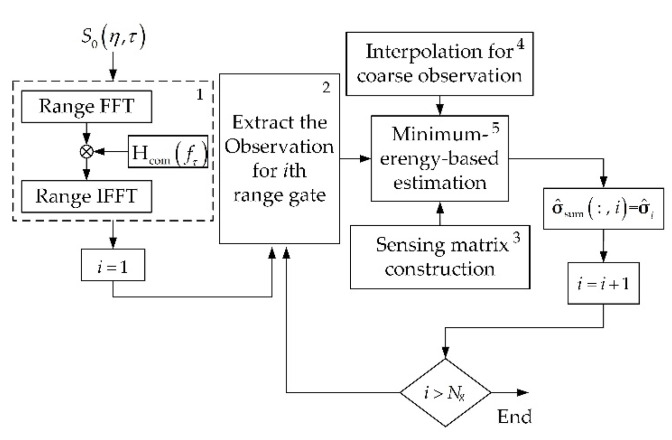
Procedure of the proposed algorithm, where $H_{\mathrm{com}}\left(f_{\tau }\right)$ is the matched filtering function, ${\hat{\sigma }}_{\mathrm{sum}}$ is the reconstructed scene, ${\hat{\sigma }}_{\mathrm{sum}}\left(\;:\;\;,\;\;i\right)={\hat{\sigma }}_{i}$ indicates that ${\hat{\sigma }}_{i}$ is assigned to the *i*th column of ${\hat{\sigma }}_{\mathrm{sum}}$, and $N_{g}$ is the number of range gates, the numbers 1–5 mark the main steps of the algorithm.

Based on Equation (8), this part proposes a minimum-energy-based algorithm that mainly includes five parts: range compression, observation vector extraction, sensing matrix construction, interpolation for coarse observation, and minimum-energy-based estimation. The procedure is shown in Figure 6, and each step is introduced as follows.

#### 3.2.1. Range Compression

The range compression filter $H_{\mathrm{com}}\left(f_{\tau }\right)$, as shown in Figure 6, can be constructed by

(9) $$H_{\mathrm{com}}\left(f_{\tau }\right)=\exp\left(-j\pi \frac{f_{\tau }^{2}}{k_{r}}\right)$$

where $f_{\tau }$ is the range frequency. After range compression, $S_{1}\left(\eta ,\tau \right)$ is obtained.

#### 3.2.2. Observation Vector Extraction

**Figure 7:**
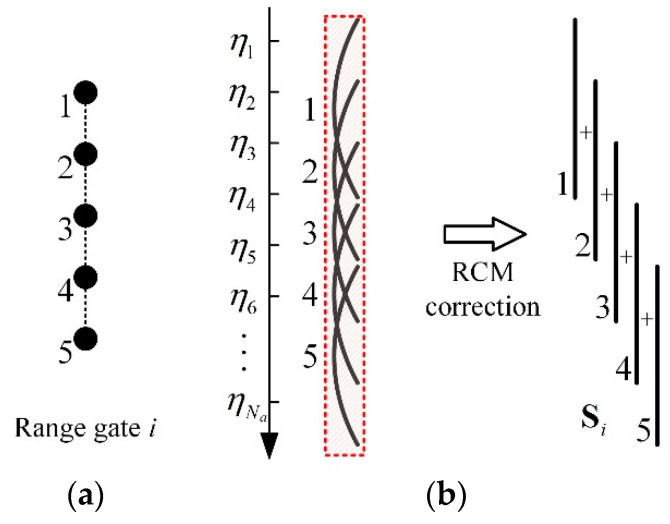
Illustration of the extraction of $S_{i}$. (**a**) Diagram of the *i*th range gate, where each point represents a pixel. (**b**) Illustration of the RCM correction of the compressed echo of the *i*th range gate.

In $S_{1}\left(\eta ,\tau \right)$, the target echoes appear as curves with range cell migration (RCM). And the RCM must be corrected to extract the observation vector $S_{i}$, which can be expressed as

(10) $$S_{i}={\left[\begin{array}{cccc} \sum_{k=1}^{K}S_{1}\left(\eta_{1},\tau_{\eta_{1},k}\right) & \sum_{k=1}^{K}S_{1}\left(\eta_{2},\tau_{\eta_{2},k}\right) & \cdots & \sum_{k=1}^{K}S_{1}\left(\eta_{N_{a}},\tau_{\eta_{N_{a}}}{}_{,k}\right) \end{array}\right]}^{T}$$

where $\tau_{\eta_{n},k}=2R\left(\eta_{n},k\right)/c$ is the main lobe’s peak position of the *k*th pixel at $\eta_{n}$. The illustration of the extraction of $S_{i}$ is shown in Figure 7.

#### 3.2.3. Observation Vector Extraction

According to the definition in Equation (5), the sensing matrix can be constructed by

(11) $$A=\left[A_{1},A_{2},\cdots ,A_{k},\cdots ,A_{K}\right]$$

with

$$A_{k}=W_{a}\left(\eta -\eta_{c,m}\right)\cdot \exp\left\{-j\frac{4\pi }{\lambda }R\left(\eta ,k\right)\right\}$$

#### 3.2.4. Interpolation for Coarse Observation

**Figure 8:**
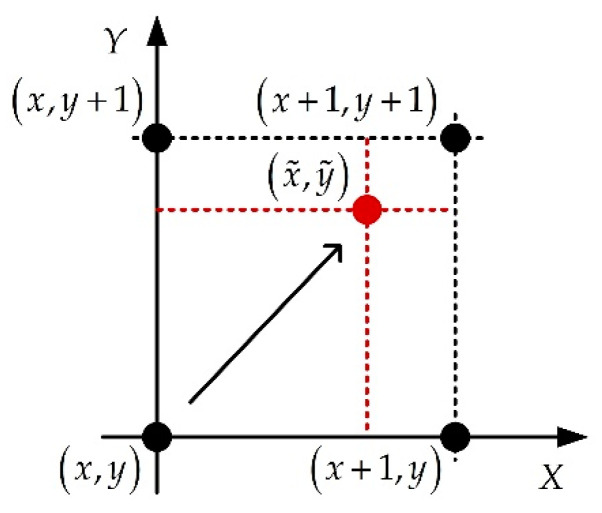
Illustration of the nearest neighbor interpolation algorithm, where (x,y) is the 2D coordinate of pixels in the original image.

Since the coarse observations from Sat-1, which are processed by the existing algorithms [1,2,3], are used as the weighting coefficients on pixels in each range gate of the final imaging result, the nearest neighbor interpolation algorithm [24] is adopted to ensure that the coarse observations are consistent with the final image’s sampling space. The interpolation principle can be expressed as

(12) $$\sigma_{d}\left(\tilde{x},\tilde{y}\right)=\sigma_{d}^{\prime }\left(\left\lceil \frac{\tilde{x}}{\Delta d_{x}/\Delta d_{x}^{\prime }}\right\rceil ,\left\lceil \frac{\tilde{y}}{\Delta d_{y}/\Delta d_{y}^{\prime }}\right\rceil \right)$$

where $\left(\tilde{x},\tilde{y}\right)$ is the 2D coordinates of pixels in the interpolated result, and $\sigma_{d}^{\prime }$ and $\sigma_{d}$ are the image amplitudes before and after the interpolation, respectively. $\Delta d_{x}$ and $\Delta d_{x}^{\prime }$ are the sampling space before and after interpolation along the X direction, respectively, and $\Delta d_{y}$ and $\Delta d_{y}^{\prime }$ are the sampling space before and after interpolation along Y direction, respectively. The diagram of interpolation is shown in Figure 8.

#### 3.2.5. Minimum-Energy-Based Estimation

To obtain the minimum weighted energy solution required in Equation (8), we define a new norm in the space consisted of band-limited sequences as

(13) $${\left\|{\hat{\sigma }}_{i}\right\|}_{\mathrm{new}}=\langle {\hat{\sigma }}_{i},Q^{-1}\cdot {\hat{\sigma }}_{i}\rangle$$

The validation of the new norm can be found in Appendix A. Then, the solution with minimum weighted energy is the solution with the smallest new norm. According to the projection theorem in [25], ${\hat{\sigma }}_{i}$ must be orthogonal in ${\langle \cdot ,\cdot \rangle }_{\mathrm{new}}$ to ℵ(A), where ℵ(A) is the null space of A and ${\langle \cdot ,\cdot \rangle }_{\mathrm{new}}$ is the new inner product corresponding to ${\left\|\;\cdot \;\right\|}_{\mathrm{new}}$. So, we obtain

(14) $${\hat{\sigma }}_{i}\in ℜ\left(A^{*}\right)$$

where $ℜ\left(A^{*}\right)$ is the range space of $A^{*}$, and $A^{*}$ is the adjoint operator of A, which can be calculated by

(15) $$A^{*}=QA^{H}$$

The specific derivation of Equation (15) can be found in the appendix of [26]. According to Equations (14) and (15), ${\hat{\sigma }}_{i}$ can be expressed as

(16) $${\hat{\sigma }}_{i}=QA^{H}\cdot v$$

where v is a $N_{a}\times 1$ vector. According to $S_{i}=A{\hat{\sigma }}_{i}$ in Equation (8), v can be calculated by

(17) $$v={\left(AQA^{H}\right)}^{-1}S_{i}$$

Then, the ${\hat{\sigma }}_{i}$ can be obtained by

(18) $${\hat{\sigma }}_{i}=QA^{H}{\left(AQA^{H}\right)}^{-1}S_{i}$$

It can be divided into the following two steps:

(19) $${\hat{\sigma }}_{\mathrm{tem}}={\left(AQA^{H}\right)}^{-1}S_{i}$$

(20) $${\hat{\sigma }}_{i}=Q\cdot A^{H}{\hat{\sigma }}_{\mathrm{tem}}$$

Step one normalizes the amplitude of the targets in $S_{i}$ as shown in Equation (19), and step two is to image the normalized result of step one as shown in Equation (20). Due to the inevitable model error and noise in the real data, we modify Equations (19)–(21) to achieve more robust imaging performance.

(21) $${\hat{\sigma }}_{\mathrm{tem}}=Q_{s}{}^{-1}S_{i}$$

where $Q_{s}$ is a diagonal matrix, its diagonal elements are $\left\{{\left|S_{i,1}\right|}^{\rho },{\left|S_{i,2}\right|}^{\rho },\cdots ,{\left|S_{i,N_{a}}\right|}^{\rho }\right\}$, and $\left|S_{i,n}\right|$ is the absolute value of the *n*th element in $S_{i}$, ρ is the normalization factor and this paper sets ρ=0.5. At the same time, Equation (20) can be modified to an iterative form, as shown in Equation (22).

(22) $${\hat{\sigma }}_{i,j}=Q_{j-1}\cdot A^{H}{\hat{\sigma }}_{\mathrm{tem}}$$

where ${\hat{\sigma }}_{i,j}$ is the *j*th reconstruction result and ${\hat{\sigma }}_{i,0}=\sigma_{d,i}$, $\sigma_{d,i}$ is the *i*th column of the coarse observation of Sat-1, $Q_{j-1}=\mathrm{diag}\left({\hat{\sigma }}_{i,j-1}\cdot {\hat{\sigma }}_{i,j-1}^{*}\right)$. This paper sets the iteration order to 2, and the detailed steps are listed in Table 1.

## 4. Verification and Analysis

This section aims to verify the discussion in the previous sections. It can be divided into three parts. Based on the SAR imaging system introduced above, Section 4.1 designs an example system and verifies the imaging performance. Section 4.2 verifies the effectiveness of the proposed algorithm with real airborne SAR data. Section 4.3 analyzes the performance variation of the proposed system with the number of subswaths. The details are as follows.

### 4.1. System Design Example and Imaging Performance

Table 2 shows the satellite parameters used in this part.

Based on the parameters in Table 2, this part designs an example HRWS-SAR system. The azimuth and range resolutions of the system are set to 3 m. The range swath is set to 200 km and divided into six subswaths. Each subswath has the same PRF and different squint angles. The beam position parameters of the two satellites are the same as in Table 3. The scanning parameters of Sat-1 are listed in Table 4.

**Figure 9:**
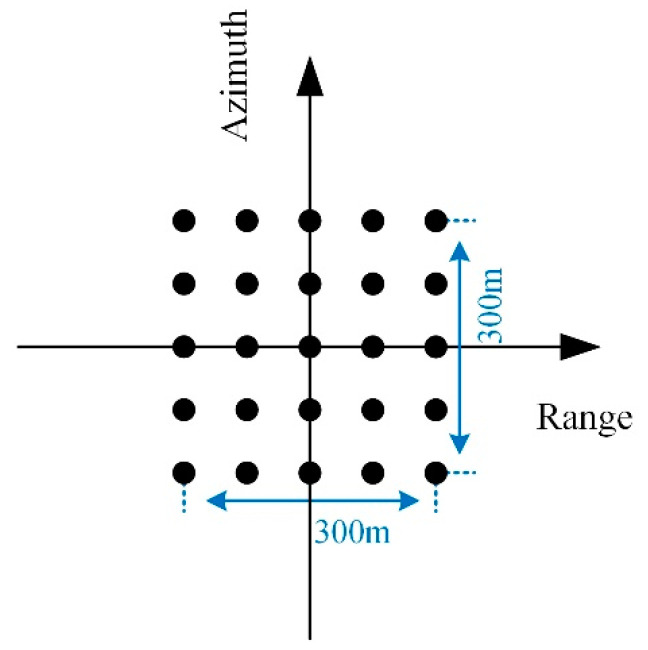
Illustration of the observed scene including 25-point targets.

**Figure 10:**
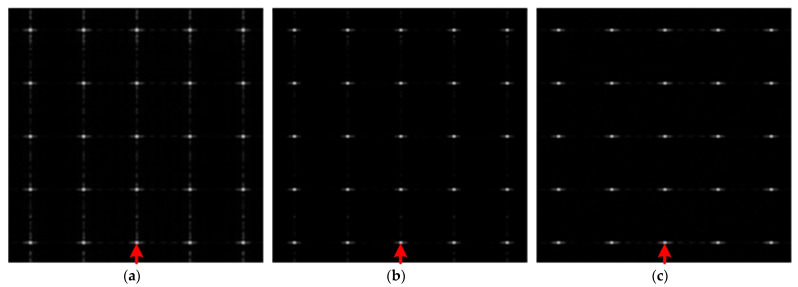
Illustration of the imaging results, where (**a**–**c**) are the results of the BP algorithm, the L1 algorithm, and the proposed algorithm, respectively.

**Figure 11:**
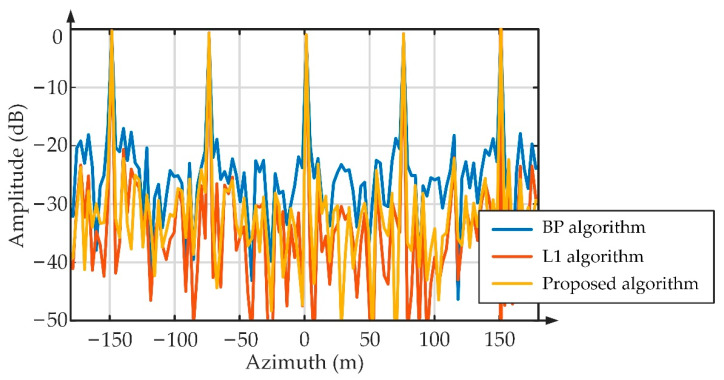
Illustration of the azimuth profile.

According to the designed SAR imaging system, the received echo of subswath 1 is simulated based on the scene as shown in Figure 9. Then, the BP algorithm, the L1 algorithm, and the proposed algorithm are carried out to image the simulated echo. According to the imaging results in Figure 10, there is obvious ambiguity energy in the imaging result of the BP algorithm, while the L1 algorithm and the proposed algorithm achieve good imaging quality. Figure 11 shows the azimuth profiles of the range gate marked by a red arrow in Figure 10.

Then, the azimuth resolution, the peak side lobe ratio, the integrated side lobe ratio, and the structure similarity are used to measure the performance of the three algorithms. The evaluated results are shown in Table 5.

Consistent with the description above, the evaluation results in Table 5 show that the proposed algorithm has the highest SSIM, PSLR, and ISLR; the imaging quality of the L1 algorithm is slightly worse; and the performance of the BP algorithm is the worst. Since the simulation scene in Figure 9 is sparse, and cannot verify the imaging performance sufficiently, the next section uses real airborne SAR data to further measure the performance of the three algorithms.

### 4.2. Verification of Real Airborne SAR Data

This section uses real airborne SAR data obtained in January 2017 in Crown Head Ridge, Guangxi, China, to verify the performance of the proposed imaging system; the parameters are listed in Table 6.

**Figure 12:**
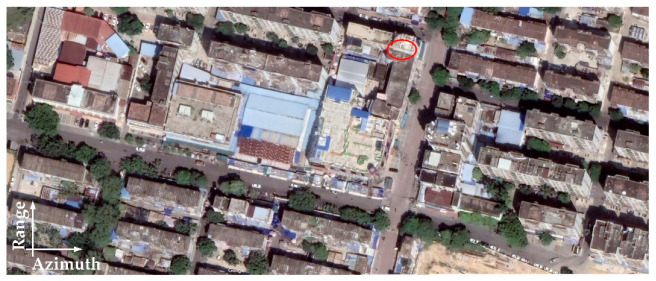
Panorama of the observed scene.

**Figure 13:**
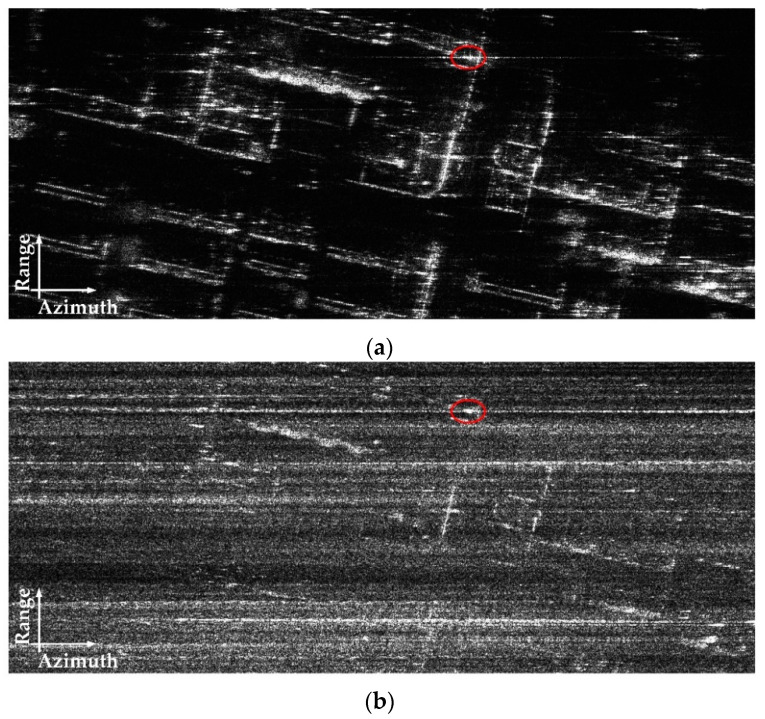
SAR images of traditional BP algorithm. (**a**) Result of original SAR data. (**b**) Result of downsampled SAR data.

The optical image of the observed scene is shown in Figure 12. The SAR imaging result processed by the traditional BP algorithm [27] is presented in Figure 13a, where a strong target is marked. Since the airborne SAR system works in strip-map mode, the original SAR data are randomly downsampled by a factor of six to construct the received echo of one subswath in the proposed system. The imaging result of the traditional BP algorithm with downsampled SAR data is presented in Figure 13b, which is seriously polluted by the ambiguity energy.

**Figure 14:**
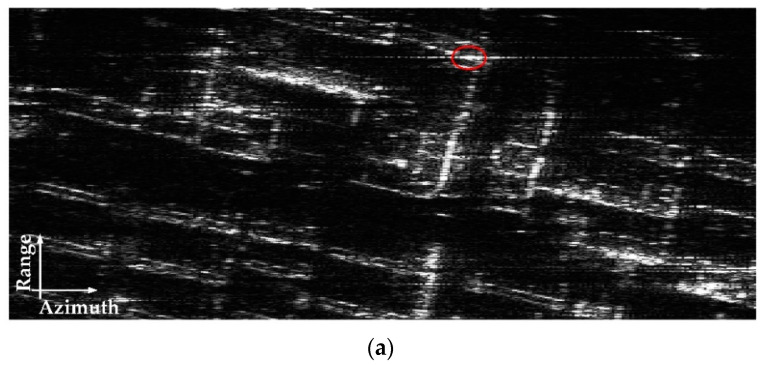

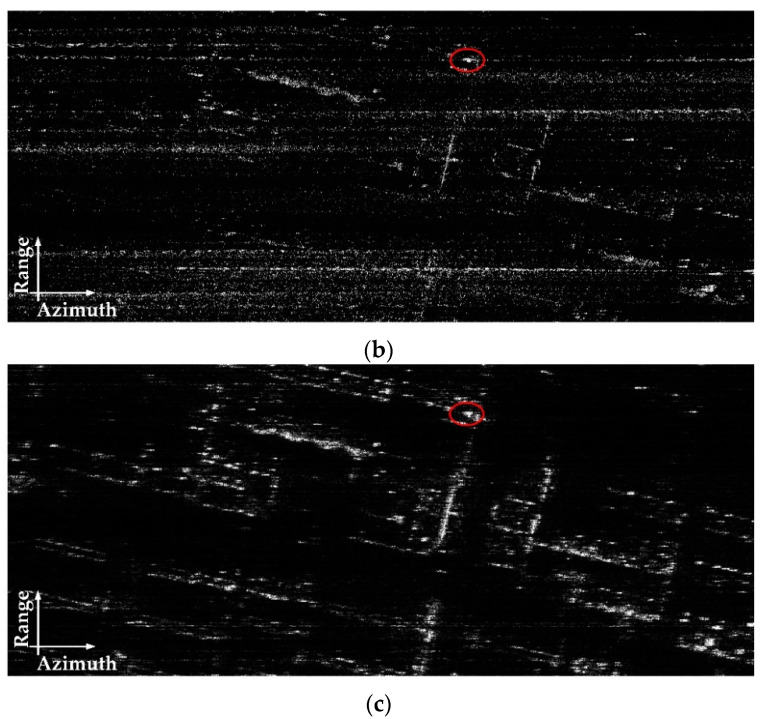
Imaging results of real airborne SAR data. (**a**) Coarse observation of Sat-1. (**b**) Reconstruction result of the L1 algorithm. (**c**) Reconstruction result of the proposed algorithm.

The result of the proposed algorithm, ${\hat{\sigma }}_{\mathrm{sum}}$, is shown in Figure 14c, and the coarse observation of Sat-1 and the result of the L1 algorithm are shown in Figure 14a,b for comparison.

According to Figure 14, the coarse observation in Figure 14a has low azimuth resolution, making the targets stack together and reducing imaging interpretation performance. The result of the L1 algorithm in Figure 14b improves the imaging resolution, but some weak target information is lost, and there are a lot of reconstruction errors in the imaging result. The proposed algorithm provides the best imaging performance, as shown in Figure 14c. It not only achieves a resolution similar to the original image as shown in Figure 13a, but also suppresses the targets’ side lobes. Then, the azimuth resolution, PSLR (peak side lobe ratio), ISLR (integral side lobe ratio), and SSIM (structure similarity index measure) are used to quantitatively evaluate the imaging quality. The calculation method of SSIM is represented in Equation (23), and all of the evaluation results are listed in Table 7.

(23) SSIM(Imx,Imy)=(2μxμy+c1)(2sxy+c2)(μx2+μy2+c1)(sx2+sy2+c2)

where Imx and Imy are the evaluated image and the reference image, respectively. $\mu_{x}$ and $\mu_{y}$ denote the average value of Imx and Imy, respectively. $s_{y}^{2}$ and $s_{x}^{2}$ denote the variance of Imx and Imy, respectively. $s_{xy}$ is the covariance of Imx and Imy.

The evaluation results in Table 6 are consistent with the imaging results in Figure 13. The L1 algorithm achieves high azimuth resolution, but the reconstruction errors lead to the deterioration of SSIM. The proposed algorithm achieves high azimuth resolution and higher sidelube suppression performance. Compared with the reference result, the proposed algorithm improves the PSLR and ISLR by 5.58 dB and 9.76 dB, respectively.

### 4.3. Analysis of the Subswath Number

According to the introduction above, Sat-2 randomly assigns the transmitted pulse to multiple subswaths. Therefore, the received echo of each subswath is undersampled, and a greater number of subswaths corresponds to fewer sampling points and worse reconstruction performance. A detailed analysis is listed in this part. The reconstruction error is regarded as noise. The signal-to-reconstruction-noise ratio is defined to analyze the reconstruction performance, that is

(24) $$SNR_{ME}=\frac{{\left\|\sigma_{i}\right\|}_{\mathrm{new}}^{2}}{{\left\|\sigma_{i}-{\hat{\sigma }}_{i}\right\|}_{\mathrm{new}}^{2}}$$

where $\sigma_{i}$ and ${\hat{\sigma }}_{i}$ are the ideal result and the estimated result of the *i*th range gate, respectively. ${\left\|\;\cdot \;\right\|}_{\mathrm{new}}$ is the new norm defined in Section 3.2.5. The ${\left\|\sigma_{i}-{\hat{\sigma }}_{i}\right\|}_{\mathrm{new}}^{2}$ in Equation (24) can be expanded as

(25) $${\left\|\sigma_{i}-{\hat{\sigma }}_{i}\right\|}_{\mathrm{new}}^{2}=\langle \left(\sigma_{i}-{\hat{\sigma }}_{i}\right),Q^{-1}\left(\sigma_{i}-{\hat{\sigma }}_{i}\right)\rangle \leq {\left\|\sigma_{i}\right\|}_{\mathrm{new}}^{2}-\sigma_{i}^{H}A^{H}{\left(AQA^{H}\right)}^{-1}A\sigma_{i}$$

Then, we can obtain

(26) $$SNR_{ME}=\frac{{\left\|\sigma_{i}\right\|}_{\mathrm{new}}^{2}}{{\left\|\sigma_{i}-{\hat{\sigma }}_{i}\right\|}_{\mathrm{new}}^{2}}\geq \frac{1}{1-\sigma_{i}^{H}A^{H}{\left(AQA^{H}\right)}^{-1}A\sigma_{i}/{\left\|\sigma_{i}\right\|}_{\mathrm{new}}^{2}}$$

**Figure 15:**
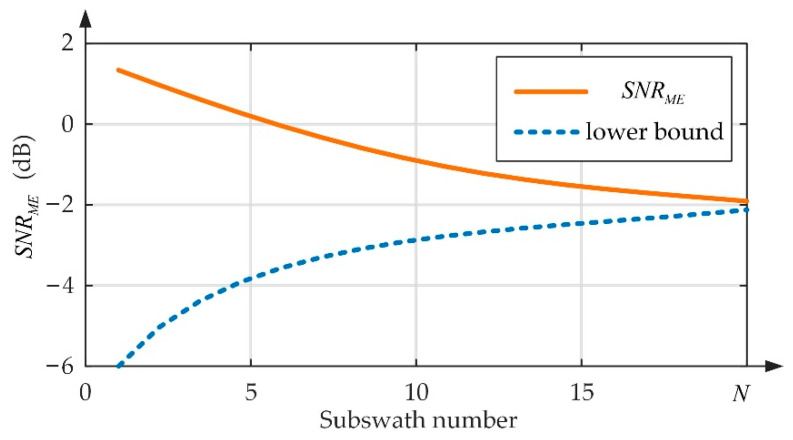
Illustration of $SNR_{ME}$ curves for different subswath numbers, where *N* is the number of subswaths.

Figure 15 shows the variation curves of $SNR_{ME}$ on the subswath number, where the lowest $SNR_{ME}$ and the highest lower bound, calculated by Equation (26), among all range gates are selected.

According to Figure 15, when the subswath number approaches 20, $SNR_{ME}$ is close to the theoretical lower bound, and the imaging result may be completely submerged by the reconstruction error.

## 5. Conclusions

In SAR observations, high resolution and wide swath contradict each other. This paper proposes a novel SAR imaging system based on tandem SAR satellites, where Sat-1 works in the traditional scanning mode to obtain coarse observations of a wide swath, and Sat-2 adopts a new scanning method to obtain the downsampled echo from the same swath. Then, a minimum-energy-based algorithm is proposed to associate the two SARs’ data and achieve high resolution. Since the accurate phase synchronization between two satellites is unnecessary, the system cost can be significantly reduced. The simulations and processed results of real airborne SAR data have proven that the proposed imaging system can expand the range swath of the strip-map mode by up to six times and achieve the high-resolution imaging of observed scenes.

## Figures and Tables

**Table 1:** Processing steps of the real airborne SAR data.

Processing Steps	Content
Step one	Obtains $S_{1}\left(\eta ,\tau \right)$ by range compression of the original echo.
Step two	Extracts the observation vectors $\left\{S_{i}|i=1,\cdots ,N_{g}\right\}$ from $S_{1}\left(\eta ,\tau \right)$
Step three	For *i* = 1: $N_{g}$ $\;\;\;\;\;\;{\hat{\sigma }}_{\mathrm{tem}}=Q_{s}{}^{-1}S_{i}$ $\;\;\;\;\;\;Q_{0}=\mathrm{diag}\left(\sigma_{d,i}\cdot \sigma_{d,i}^{*}\right)$ $\;\;\;\;\;\;{\hat{\sigma }}_{i,1}=Q_{0}\cdot A^{H}{\hat{\sigma }}_{\mathrm{tem}}$ $\;\;\;\;\;\;Q_{1}=\mathrm{diag}\left({\hat{\sigma }}_{i,1}\cdot {\hat{\sigma }}_{i,1}^{*}\right)$ $\;\;\;\;\;\;{\hat{\sigma }}_{i,2}=Q_{1}\cdot A^{H}{\hat{\sigma }}_{\mathrm{tem}}$ End
Step four	Output: $\;\;\;\;\;\;{\hat{\sigma }}_{\mathrm{sum}}=\left[\begin{array}{cccc} {\hat{\sigma }}_{1,2} & {\hat{\sigma }}_{2,2} & \cdots & {\hat{\sigma }}_{N_{g},2} \end{array}\right]$

**Table 2:** System parameters for Sat-1 and Sat-2.

Parameters	Value
Carrier frequency (GHz)	9.6
Range bandwidth (MHz)	100
Sampling frequency (MHz)	120
Pulse width (µs)	20
Orbital height (km)	630
Platform velocity (m/s)	7545
Resolution (m)	3

**Table 3:** Beam position parameters of six subswaths.

	Look Angle in the Zero Doppler Plane (°)	PRF (Hz)	Squint Angle (°)	Subswath Width (km)
Subswath 1	32.01~34.41°	2673	0°	39.90
Subswath 2	29.79~32.19°	2673	8.70°	37.58
Subswath 3	27.51~29.91°	2673	15.30°	35.55
Subswath 4	25.23~27.63°	2673	19.40°	33.82
Subswath 5	22.91~25.31°	2673	22.50°	32.31
Subswath 6	20.55~22.95°	2673	26.80°	31.00

**Table 4:** Scanning parameters of Sat-1.

	Burst Duration(s)	3 dB Doppler Bandwidth (MHz)
Subswath 1	0.077	2446
Subswath 2	0.075	2428
Subswath 3	0.074	2323
Subswath 4	0.072	2231
Subswath 5	0.070	2148
Subswath 6	0.069	2032

**Table 5:** Evaluation results.

	BP Algorithm	L1 Algorithm	Proposed Algorithm
Azimuth resolution (m)	2.94	2.73	2.73
PSLR (dB)	−16.96	−20.55	−22.62
ISLR (dB)	−9.64	−15.16	−15.74
SSIM	0.61	0.80	0.81

**Table 6:** System parameters for airborne SAR.

Parameters	Value
Carrier frequency (GHz)	35
Range bandwidth (MHz)	480
Sampling frequency (MHz)	500
Pulse width (µs)	25
Slant range (km)	27.21
Velocity (m/s)	90.86
Doppler bandwidth (MHz)	600
Duration time (s)	15

**Table 7:** Evaluation results.

	Reference	Coarse Observation	L1 Algorithm	Proposed Algorithm
Azimuth resolution (m)	0.6	3.6	0.6	0.6
PSLR (dB)	−12.27	−11.77	−10.47	−17.84
ISLR (dB)	−7.37	−9.35	−7.97	−17.13
SSIM	1	0.89	0.87	0.93

## Appendix A

Given a vector space X over a subfield F of the complex numbers ℂ, a norm on X is a real-valued function ‖ ⋅ ‖: X→ℝ with the following properties, where |a| denotes the usual absolute value of a scalar a:

(1) Triangle inequality: ‖x+y‖≤‖x‖+‖y‖ for all x,y∈X.
(2) Absolute homogeneity: ‖ax‖=|a|⋅‖x‖ for all x∈X and all scalar a.
(3) Positive definiteness: for all x∈X, ‖x‖≥0 and ‖x‖=0⇔x=0.

Then, the validation of the new norm ${\left\|\;\cdot \;\right\|}_{\mathrm{new}}$ defined in Equation (13) is derived as follows. According to the definition, ${\left\|x\right\|}_{\mathrm{new}}$ can be calculated by

(A1) $${\left\|x\right\|}_{\mathrm{new}}=\sqrt{\langle x,Q^{-1}x\rangle }=\sqrt{\sum_{n}q_{nn}^{-1}\cdot {\bar{x}}_{n}x_{n}}$$

where $x_{n}$ is the *n*th element of x, and $q_{nn}$ is the *n*th row and *n*th column element in Q. Since ${\bar{x}}_{n}x_{n}>0$ and $q_{nn}^{-1}>0$, the positive definiteness of ${\left\|x\right\|}_{\mathrm{new}}$ is easily proved. Then, according to Equation (A1), we have

(A2) $$\begin{array}{l} {\left\|x+y\right\|}_{\mathrm{new}}=\sqrt{\sum_{n}q_{nn}^{-1}\cdot \left({\bar{x}}_{n}+{\bar{y}}_{n}\right)\left(x_{n}+y_{n}\right)}\\ \;\;\;\;=\sqrt{\sum_{n}q_{nn}^{-1}\cdot {\bar{x}}_{n}x_{n}+\sum_{n}q_{nn}^{-1}\cdot {\bar{x}}_{n}y_{n}+\sum_{n}q_{nn}^{-1}\cdot {\bar{y}}_{n}x_{n}+\sum_{n}q_{nn}^{-1}\cdot {\bar{y}}_{n}y_{n}}\\ \;\;\;\;\leq \sqrt{\sum_{n}q_{nn}^{-1}\cdot {\bar{x}}_{n}x_{n}+\sum_{n}q_{nn}^{-1}\cdot {\bar{y}}_{n}y_{n}+2\sqrt{\sum_{n}q_{nn}^{-1}\cdot {\bar{x}}_{n}x_{n}}\sqrt{\sum_{n}q_{nn}^{-1}\cdot {\bar{y}}_{n}y_{n}}}\\ \;\;\;\;={\left\|x\right\|}_{\mathrm{new}}+{\left\|y\right\|}_{\mathrm{new}} \end{array}$$

and

(A3) $${\left\|ax\right\|}_{\mathrm{new}}=\sqrt{\sum_{n}q_{nn}^{-1}\cdot a{\bar{x}}_{n}ax_{n}}=\left|a\right|\cdot \sqrt{\sum_{n}q_{nn}^{-1}\cdot {\bar{x}}_{n}x_{n}}=\left|a\right|\cdot {\left\|x\right\|}_{\mathrm{new}}$$

So ${\left\|\;\cdot \;\right\|}_{\mathrm{new}}$ satisfies property 1 and 2. Then, the validation of ${\left\|\;\cdot \;\right\|}_{\mathrm{new}}$ is proven.

## References

[1] An D., Huang X., Jin T. (2012). Extended Nonlinear Chirp Scaling Algorithm for High-Resolution Highly Squint SAR Data Focusing. IEEE Trans. Geosci. Remote Sens..

[2] Xu W., Deng Y., Huang P. (2014). Full-Aperture SAR Data Focusing in the Spaceborne Squinted Sliding-Spotlight Mode. IEEE Trans. Geosci. Remote Sens..

[3] Sun G., Wu Y., Yang J. (2017). Full-Aperture Focusing of Very High Resolution Spaceborne-Squinted Sliding Spotlight SAR Data. IEEE Trans. Geosci. Remote Sens..

[4] Krieger G., Gebert N., Moreira A. (2008). Multidimensional waveform encoding: A new digital beamforming technique for synthetic aperture radar remote sensing. IEEE Trans. Geosci. Remote Sens..

[5] Hu C., Li Y., Dong X. (2017). Performance Analysis of L-Band Geosynchronous SAR Imaging in the Presence of Ionospheric Scintillation. IEEE Trans. Geosci. Remote Sens..

[6] Dong X., Quegan S., Yumiko U., Hu C., Zeng T. (2015). Feasibility Study of C- and L-band SAR Time Series Data in Tracking Indonesian Plantation and Natural Forest Cover Changes. IEEE J. Sel. Top. Appl. Earth Obs. Remote Sens..

[7] Freeman A., Johnson W.T.K., Huneycutt B. (2000). The “Myth” of the minimum SAR antenna area constraint. IEEE Trans. Geosci. Remote Sens..

[8] Zhang Y., Wang W., Deng Y., Wang R. (2020). Signal Reconstruction Algorithm for Azimuth Multichannel SAR System Based on a Multiobjective Optimization Model. IEEE Trans. Geosci. Remote Sens..

[9] Chen Z., Zhang Z., Zhou Y. (2022). Elevated Frequency Diversity Array: A Novel Approach to High Resolution and Wide Swath Imaging for Synthetic Aperture Radar. IEEE Geosci. Remote Sens. Lett..

[10] Krieger G., Huber S., Villano M. SIMO and MIMO System Architectures and Modes for High-Resolution Ultra-Wide-Swath SAR Imaging. Proceedings of the EUSAR: European Conference on Synthetic Aperture Radar.

[11] Liu B., He Y. (2016). Improved DBF Algorithm for Multichannel High-Resolution Wide-Swath SAR. IEEE Trans. Geosci. Remote Sens..

[12] Sun L., Yu Z., Li C. (2022). A Minimum-Energy-Based Algorithm for Multichannel Reconstruction. IEEE Geosci. Remote Sens. Lett..

[13] Xia Z., Zhao Z., Zhang T. Wide-Swath SAR Based on Networking of Multiple Small Satellites for Maritime Applications. Proceedings of the IET International Radar Conference.

[14] Krieger G., Moreira A. (2006). Spaceborne bi- and multistatic SAR: Potential and challenges. IEE Proc. Radar Sonar Navig..

[15] Zhang Y., Zhang H., Hou S. (2021). An Innovative Superpolyhedron (SP) Formation for Multistatic SAR (M-SAR) Interferometry. IEEE Trans. Geosci. Remote Sens..

[16] Krieger G., Zonno M., Mittermayer J. MirrorSAR: A Fractionated Space Transponder Concept for the Implementation of Low-Cost Multistatic SAR Missions. Proceedings of the Eusar: European Conference on Synthetic Aperture Radar.

[17] Chen Y., Zhao Y., Li G. High-Resolution and Wide-Swath Monostatic SAR Imaging via Random Beam Scanning. Proceedings of the 2020 IEEE 11th Sensor Array and Multichannel Signal Processing Workshop.

[18] Gebert N., Krieger G., Moreira A. (2010). Multichannel Azimuth Processing in ScanSAR and TOPS Mode Operation. IEEE Trans. Geosci. Remote Sens..

[19] Huang L., Qiu X., Hu D. (2015). Medium-Earth-Orbit SAR Focusing Using Range Doppler Algorithm With Integrated Two-Step Azimuth Perturbation. IEEE Geosci. Remote Sens. Lett..

[20] Cumming I.G., Wong F.H. (2005). Digital Processing of Synthetic Aperture Radar Data: Algorithms and Implementation.

[21] Lao D., Zhu B., Yu S. An Improved SAR Imaging Algorithm Based on a Two-Dimension-Separated Algorithm. Proceedings of the 2018 China International SAR Symposium (CISS).

[22] Li D., Lin H., Liu H. (2017). Focus Improvement for High-Resolution Highly Squinted SAR Imaging Based on 2-D Spatial-Variant Linear and Quadratic RCMs Correction and Azimuth-Dependent Doppler Equalization. IEEE J. Sel. Top. Appl. Earth Obs. Remote Sens..

[23] Papoulis A. (1975). A new algorithm in spectral analysis and band-limited extrapolation. IEEE Trans. Circuits Syst..

[24] Parker J.A., Kenyon R.V., Troxel D.E. (1983). Comparison of Interpolating Methods for Image Resampling. IEEE Trans. Med. Imaging.

[25] Luenberger D.G. (1969). Optimization by Vector Space Methods.

[26] Potter L.C., Arun K.S. (1989). Energy concentration in band-limited extrapolation. IEEE Trans. Acoust. Speech Signal Process..

[27] Sahiner B., Yagle A.E. (1993). A fast algorithm for backprojection with linear interpolation. IEEE Trans. Image Process..

